# Acute EBV infection masquerading as "In-situ Follicular Lymphoma": a pitfall in the differential diagnosis of this entity

**DOI:** 10.1186/1746-1596-8-100

**Published:** 2013-06-19

**Authors:** Alejandro A Gru, Friederike Kreisel, Eric Duncavage, TuDung T Nguyen, Anjum Hassan, John L Frater

**Affiliations:** 1 Department of Pathology, The Ohio State University Wexner Medical Center, Columbus, OH, USA; 2 Department of Pathology and Immunology, Washington University School of Medicine, 660 S. Euclid Avenue, Box 8118, St. Louis, MO 63110, USA

## Abstract

We present the case of a 30 year-old man who was referred for evaluation of diffuse lymphadenopathy. Six weeks prior, he noticed darkening of his urine associated with pale stools, nausea and an eventual 30 lb weight loss within a month. The initial laboratory findings showed elevation of the liver enzymes. A CT scan showed mesenteric and periaortic lymphadenopathy with the largest lymph node measuring 2.8 cm. Other laboratory results were otherwise unremarkable (including a normal LDH) with the exception of positive serum antibodies against Epstein-Barr virus (EBV) associated antigens (IgM+ and IgG+). An excisional biopsy of 4 of the small neck lymph nodes showed a normal architecture with prominent follicles and an intact capsule. But, by immunohistochemistry two of the follicles showed aberrant coexpression of BCL-2, in addition to CD10 and BCL-6. In-situ hybridization for early Epstein-Barr virus mRNA (EBER) and immunohistochemistry for latent membrane protein-1 (LMP-1) stained both scattered positive cells, as well as BCL-2 positive B-cells. Although an original diagnosis of in-situ follicular lymphoma was favored at an outside facility, additional interphase fluorescence in situ hybridization (FISH) studies for t(14;18);(*IGH-BCL2*) rearrangement (performed on the BCL-2 + follicles microdissected from the tissue block; Abott probe dual colour fusion) and molecular studies (IGH gene rearrangement by PCR, also performed on the microdissected follicles) were negative. Serologic studies (positive EBV antibodies) and immunostains in conjunction with the molecular studies confirmed the reactive nature of the changes. Our case also shows direct immunopathogenic evidence of BCL-2 expression among the EBV-infected cells, which has to our knowledge not been previously documented *in vivo.* A diagnosis of EBV infection should, therefore, be considered when confronted with BCL-2 expression in germinal centers, particularly in younger individuals, as the diagnosis of FLIS may lead to extensive and invasive haematologic work-ups.

**Virtual slides:**

The virtual slide(s) for this article can be found here: http://www.diagnosticpathology.diagnomx.eu/vs/1323656318940068

## Case report

A 30 year-old man was referred for evaluation of diffuse lymphadenopathy. 6 weeks prior, the patient noticed darkening of his urine associated with pale stools, nausea and an eventual 30 lb weight loss within a month. He also complained of fever, myalgias, joint pain, and fatigue, which occurred approximately 48 hours after the onset of the urine colour changes. The initial laboratory results showed elevation of liver enzymes (AST 278 Units/L, ALT 831 Units/L and total bilirubin of 1.9 mg/dl). The complete blood count (CBC) included the following results: WBC 8.4 (neutrophils 54.5%, lymphocytes 34.3%, monocytes 7.8%, eosinophils 2.5% and basophils 0.9%), Hgb 15.9, hematocrit 47.3, platelet count 151, LDH 179, RBC 5.12 MCV 92.5 and RDW 13.2. An abdominal ultrasound revealed a 2.9 cm mass within the pancreas and the liver. A follow-up CT scan showed mesenteric and periaortic lymphadenopathy with the largest lymph node measuring 2.8 cm. Two weeks later, the majority of the symptoms resolved, but the patient noticed new enlarged lymph nodes in the right neck and in the left groin, measuring less than 1 cm. No associated hepatosplenomegaly was identified. The patient's admission laboratory results were otherwise unremarkable (including a normal LDH) with the exception of positive serum antibodies against Epstein-Barr virus (EBV) associated antigens (IgM+ and IgG+).

**Figure 1:**
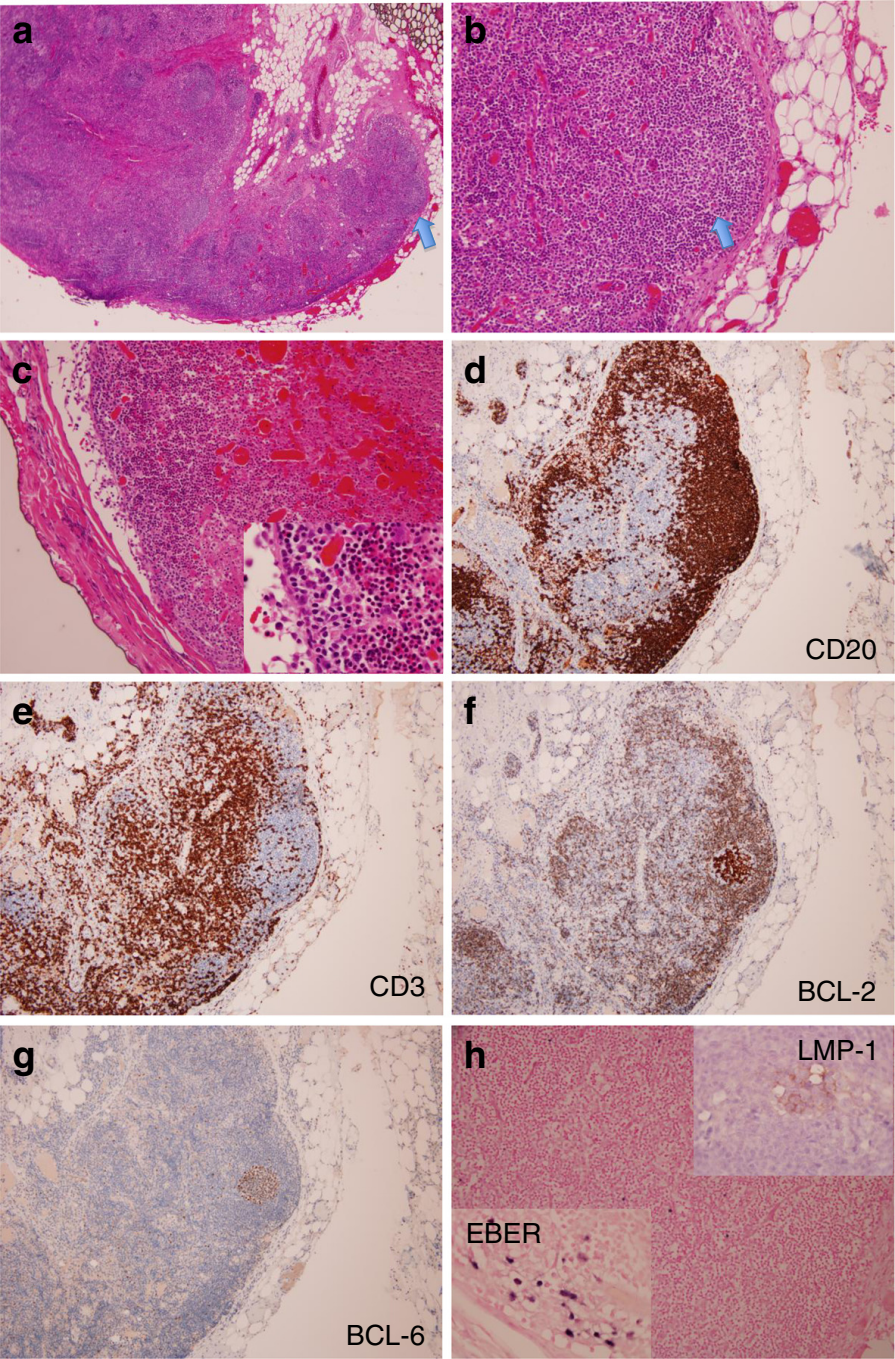
**Excisional (1st) biopsy from cervical lymph node. a** and **b** – low power view. Reactive appearing follicles with preserved architecture. **c** – infarcted lymph node with occasional large cells with prominent nucleoli on high magnification. **d** – CD20. **e** – CD3. **f** – BCL-2. **g** – BCL-6. **h** – EBER and LMP-1.

**Figure 2:**
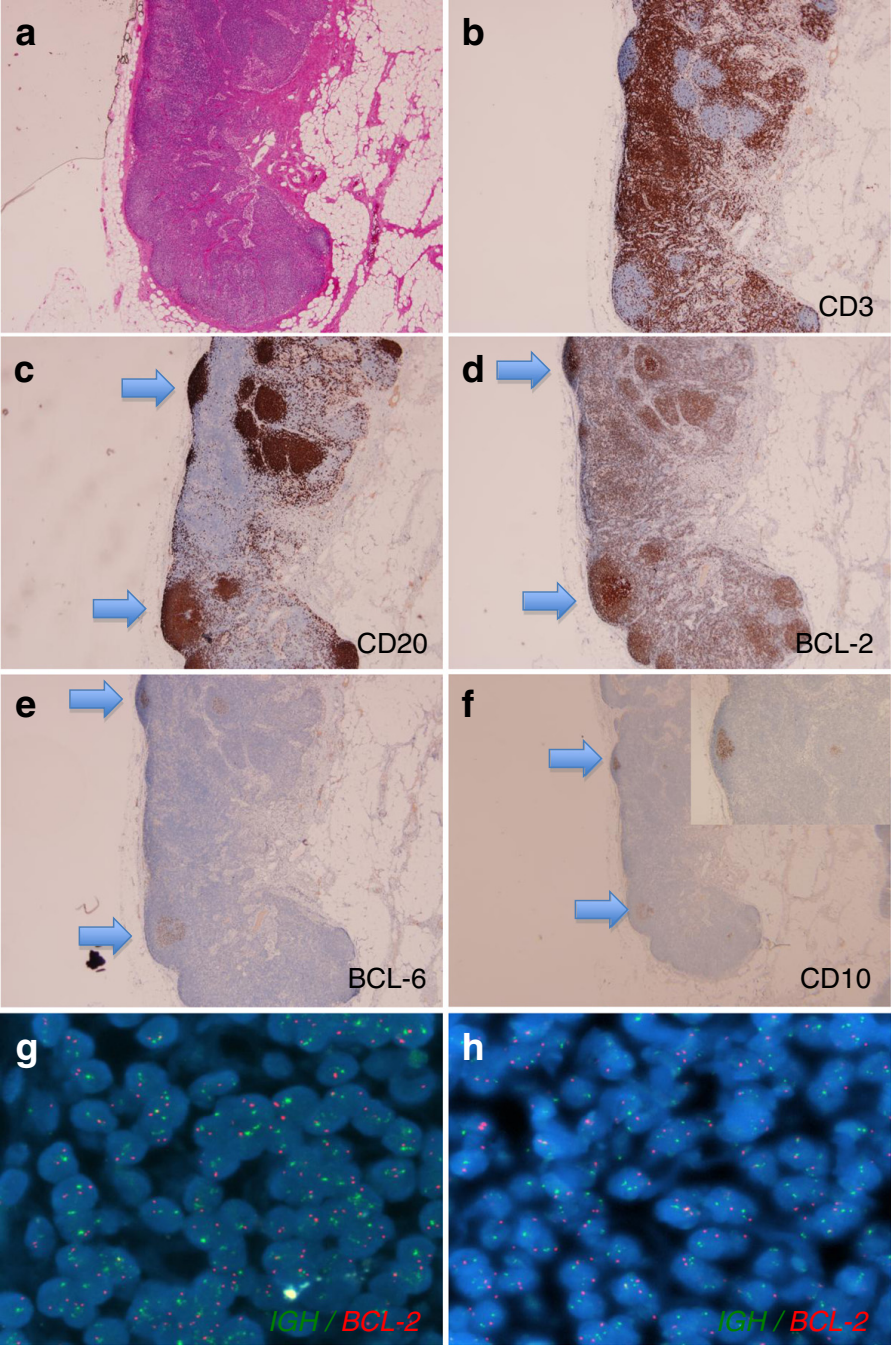
**Excisional (2nd) biopsy from inguinal lymph node. a** – lymph node, second biopsy at low power showing similar morphologic findings. **b** – CD3. **c** - CD20. **d** – BCL-2. **e** – BCL-6. **f** – CD10. The arrows indicate the abnormal phenotype in some of the follicles. **g** and **h** – FISH using Vysis dual color-fusion probes for IGH-BCL-2 with no evidence of rearrangement.

An excisional biopsy of 4 of the small neck lymph nodes showed a normal architecture with prominent follicles (Figure 1) and an intact capsule. Two of the lymph nodes appeared to have changes that were suggestive of infarction and/or hemorrhage. In the subcapsular space a group of larger cells with coarser chromatin and more prominent nucleoli was seen. Immunohistochemistry showed reactive appearing CD20-positive follicles with interfollicular CD3-positive T-cells. Two of the follicles showed aberrant coexpression of BCL-2, in addition to CD10 and BCL-6. A subsequent biopsy of inguinal lymph nodes (Figure 2) showed similar morphologic changes with approximately 3–4 additional follicles revealing abnormal BCL-2 coexpression among the B-cells with a germinal center phenotype. In-situ hybridization for early Epstein-Barr virus mRNA (EBER) and immunohistochemistry for latent membrane protein-1 (LMP-1) stained both scattered positive cells, as well as BCL-2 positive B-cells. Although an original diagnosis of in-situ follicular lymphoma was favored at an outside facility, additional interphase fluorescence in situ hybridization (FISH) studies for t(14;18);(*IGH-BCL2*) rearrangement (performed on the BCL-2 + follicles microdissected from the tissue block; Abott probe dual colour fusion) and molecular studies (IGH gene rearrangement by PCR, also performed on the microdissected follicles) were negative. Serologic studies (positive EBV antibodies) and immunostains in conjunction with the molecular studies confirmed the reactive nature of the changes.

The patient's findings have completely resolved, and there is no evidence of lymphoma after 9 months of follow-up.

## Discussion

This case represents an unusual presentation of infectious mononucleosis with immunophenotypic findings suggestive of in-situ follicular lymphoma. This observation has not, to our knowledge, been previously reported in the English-language medical literature. In 2002 Jaffe et al. [1] described 25 patients with lymphadenopathy having abnormal BCL-2 overexpression in follicle centers, associated with fairly preserved tissue architecture and residual reactive germinal centers. The term in-situ follicular lymphoma (FLIS) was coined to describe such lesions, and was subsequently incorporated into the World Health Organization classification of hematopoietic neoplasms. A recent study showed that FLIS typically occurs in the 5th or 6th decade of life, with only rare cases reported in patients under 40 years of age. The follicular lymphoma associated t(14;18)(*IGH;BCL2*) translocation is routinely demonstrated in the vast majority of the abnormal follicles. Patients with FLIS were originally reported as having a very low risk for developing overt follicular lymphoma [2]. Others have suggested that FLIS indicates a possible increased risk of several forms of lymphoid neoplasia, though many patients remain free of malignancy after an extensive follow-up and staging [3,4]. The diagnosis of FLIS is typically an incidental finding, where lymph nodes are biopsied secondary to reactive follicular hyperplasia or, sometimes, other pathology. To date, very little attention has been paid to potential pathologic mimics of FLIS, although some have shown that FLIS can be seen in association with progressive transformation of germinal centers [5,6].

Epstein-Barr virus (EBV) is a human herpesvirus with an overall seroprevalence of >90% in all adults. It is thought that in early infection, EBV-infected cells undergo large-scale expansion within the germinal centers. However, the total number of EBV-positive cells even in the acute phase of the disease is low, as previously shown in germinal centers of tonsils from patients with infectious mononucleosis. The EBV infected cells show expression of BCL-6 and CD10, common germinal center markers with variable staining for LMP-1, LMP-2 and EBNA-1, which are EBV latent proteins. EBV also induces upregulation and overexpression of BCL-2 among the B-cells, findings commonly observed in latent EBV infection but also in carcinomas which are EBV positve and EBV+ large B-cell lymphomas [7,8]. In addition, in experimental cell lines transfected with EBV, LMP-1 was capable of NF-κβ activation leading to BCL-2 over-expression.

The EBV virus has a main pathogenic role in the development of lymphoid and non-lymphoid malignancies [9,10]. Several studies have shown its association with specific entities such as post-transplant lymphoproliferative disorders of both B and T-cell lineage. Additionally, EBV has a main role in the development of certain B-cell lymphomas, such as endemic Burkitt's lymphoma, certain types of classical Hodgkin lymphoma, EBV-positive diffuse large B-cell lymphoma (DLCBL) of the elderly, DLBCL associated with chronic inflammation and lymphomatoid granulomatosis (among others). It is also related to T/NK malignancies which include aggressive NK cell leukemia, EBV-positive T-cell lymphoproliferative disorders of childhood, extranodal NK/T-cell lymphoma and angioimmunoblastic T-cell lymphomas [11].

The diagnosis of FLIS is most frequently made in patients who are 50–60 years of age, and older than the reported case. In addition, the serologic findings were confirmatory for the diagnosis of infectious mononucleosis. Even though the immunophenotypic findings were suggestive of FLIS, the absence of a t(14;18) rearrangement, and the presence of EBER and LMP-1 expression were also helpful to exclude FLIS. Some authors have also suggested that the pattern of intensity for CD10 (usually stronger in FLIS) can help distinguish FLIS from other mimickers [12]. In our case, the CD10 intensity was similar in both the BCL-2 coexpressing and non-coexpressing follicles. Further, the patient developed no subsequent lymphadenopathy and had complete resolution of his symptoms. Our case also shows direct immunopathogenic evidence of BCL-2 expression among the EBV-infected cells, which has to our knowledge not been previously documented *in vivo.* A diagnosis of EBV infection should, therefore, be considered when confronted with BCL-2 expression in germinal centers, particularly in younger individuals, as the diagnosis of FLIS may lead to extensive and invasive haematologic work-ups.

### Consent

Written informed consent was obtained from the patient for publication of this case report and any accompanying image.

## Competing interests

The authors declare that they have no competing interests.

## Authors’ contributions

AAG was the main author on the paper, took the clinical images, worked up the case, wrote the manuscript and performed adequate corrections. FK proofread the text and made suggestions for corrections in the body of the manuscript. ED proofread the text and made corrections in the body of the manuscript. TN proofread the text and made corrections in the body of the manuscript. AH proofread the text and made corrections in the body of the manuscript. JLF was the main pathologist involved in the case, also collaborated in writing the manuscript, was the main editor of the body of the text, and also participated in obtaining the clinical images. All authors read and approved the final manuscript.
